# Nanomaterials as a Sustainable Choice for Treating Wastewater: A Review

**DOI:** 10.3390/ma15238576

**Published:** 2022-12-01

**Authors:** Wael Ben Mbarek, Lluisa Escoda, Joan Saurina, Eloi Pineda, Fahad M. Alminderej, Mohamed Khitouni, Joan-Josep Suñol

**Affiliations:** 1 Department of Physics, Campus Montilivi s/n, University of Girona, 17003 Girona, Spain; 2 Department of Physics, Institute of Energy Technologies, Universitat Politècnica de Catalunya, 08019 Barcelona, Spain; 3 Department of Chemistry, College of Science, Qassim University, Buraidah 51452, Saudi Arabia

**Keywords:** nanocatalysts, nanomembranes, nanoadsorbents, wastewater treatment, nanomaterial challenges

## Abstract

The removal of dyes from textile effluents utilizing advanced wastewater treatment methods with high efficiency and low cost has received substantial attention due to the rise in pollutants in water. The purpose of this work is to give a comprehensive analysis of the different treatments for removing chemical dyes from textile effluents. The capability and potential of conventional treatments for the degradation of dyeing compounds in aqueous media, as well as the influence of multiple parameters, such as the pH solution, initial dye concentration, and adsorbent dose, are presented in this study. This study is an overview of the scientific research literature on this topic, including nanoreductive and nanophotocatalyst processes, as well as nanoadsorbents and nanomembranes. For the purpose of treating sewage, the special properties of nanoparticles are currently being carefully researched. The ability of nanomaterials to remove organic matter, fungus, and viruses from wastewater is another benefit. Nanomaterials are employed in advanced oxidation techniques to clean wastewater. Additionally, because of their small dimensions, nanoparticles have a wide effective area of contact. Due to this, nanoparticles’ adsorption and reactivity are powerful. The improvement of nanomaterial technology will be beneficial for the treatment of wastewater. This report also offers a thorough review of the distinctive properties of nanomaterials used in wastewater treatment, as well as their appropriate application and future possibilities. Since only a few types of nanomaterials have been produced, it is also important to focus on their technological feasibility in addition to their economic feasibility. According to this study, nanoparticles (NPs) have a significant adsorption area, efficient chemical reactions, and electrical conductivity that help treat wastewater effectively.

## 1. Introduction

Dyed effluents are of great environmental concern. Textile industries consume a large amount of water and produce a remarkable amount of wastewater containing pigments, dyes, and non-expendable components. Dyes give off their color by staining or being absorbed after their dissolution in specific solutions. Various atomic groups, known as chromophores, determine whether an organic source seems to be a dye or not. The azo group, thio group, nitroso group, carbonyl group, nitro group, and azoxy group are examples of chromophores. Auxochromes are different groups of atoms that link to dye molecules, contribute or receive electrons, improve color, and boost solubility. Auxochrome groups consist of substituted amino, hydroxyl, sulfonic, and amino groups. Dyes are classified depending on how well they color. There are various dyes used, and even more textiles and materials now contain colorants during production. Depending on the chemical characteristics of the dye and the physical characteristics of the material to be dyed, or the dyeing properties, certain dyes are employed for specific materials. Basic or cationic, acid and premetalized, chrome and mordant, direct, sulfur, dispersion, vat, azoic, and reactive dyes are the different categories of dyeing characteristics.

The generation of wastewater that is highly polluted and contains dyes is a very serious problem for sewage treatment stations. In addition, there are some highly toxic materials and textile waste products that are responsible for reducing the ability of self-decomposition of pollutants in wastewater [1]. Physical, chemical, and biological approaches are three that are frequently used to address the degradation of residual azo dyes in wastewater. Examples of physical and chemical treatments include adsorption, flocculation, electrocoagulation, precipitation, ozonation, and irradiation. However, these procedures may be not enough to eradicate dyes from wastewater. For example, the biological method is not suitable for eliminating color from dyes because most of them are inorganic and toxic to the microorganisms used in the process. However, physical techniques, such as membrane filtering, ion exchange, and adsorption, have key limitations: they work best when the volume of wastewater is modest and can be further broken down into a few tiny components that are challenging to digest. During the molecular process of adsorption, forces of attraction bind a solute (adsorbate) to a solid surface (adsorbent). The coagulation–flocculation procedure is widely used as a pretreatment stage to improve the effectiveness of subsequent processes, such as sedimentation and filtration, in a water treatment system. In the coagulation process, the coagulant is primarily responsible for destabilizing the colloidal particles. The flocculation process, which increases the solution’s unstable particle size into larger flocs, comes next. This technique enables the removal of suspended solids and colloidal particles from the solution. Large amounts of organic contaminants can be effectively removed using the coagulation and flocculation process [2]. In addition, researchers have been very interested in plasma technology for a variety of environmental applications [3]. Pesticides in wastewater can be detoxified and degraded using cold (non-thermal) plasma technology, which does not create any secondary pollutants in the process [4]. Due to the creation of UV radiation, shock waves, and highly reactive species that oxidize and mineralize the contaminants into CO_2_, H_2_O, and simpler inorganics, cold plasma is also a well-known technique for cleaning water.

In order to change the phases, physical treatments, such as filtration, can be utilized to eliminate the contaminants. However, this modification leads to the release of a significant amount of sludge, which is hazardous to the environment and difficult to dispose of [5,6]. In addition, most dyes exacerbate the environmental problem due to the formation of undesirable decomposition products [7]. The degradation of dyes from textile waste can be very difficult due to their complex structure [8].

**Figure 1:**
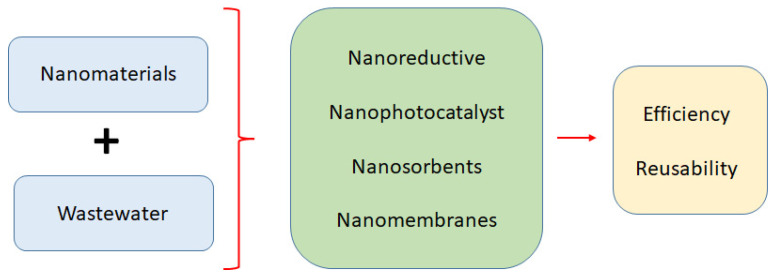
Nanomaterials and processes in the wastewater treatment.

To the best of our knowledge, despite the wide range of studies in this field, there are not many review articles that have discussed the use of nanomaterials in the degradation of organic dyes in wastewater. Throughout this review paper, we have attempted to investigate the synergistic impact of using nanomaterials in the degradation of organic pollutants as well as discuss their efficacy in the reduction of contaminants and the reuse of wastewater in industrial cycles. The most suitable technology to effectively and economically eliminate contaminants is nanotechnology, which is seen as an emerging discipline that offers an alternative [9]. The nanomaterials range in size from a few nanometers to less than 100 nm [10]. Occasionally, specimens with nanostructures are regarded as nanomaterials. Around the world, there are several environmental problems that nanomaterials can potentially solve. Nanomaterials come in a variety of forms, including nanowires, nanotubes, films, particles, colloids, and quantum dots [11]. Nanophotocatalysts, nanoreductives, nanomembranes, and nanosorbents are the four main types of nanomaterial that can play a crucial part in the wastewater treatment process (Figure 1). The distinctive attributes of nanomaterials, such as their high surface area, large pores, high reactivity, strong mechanical properties, ease of dispersion, and hydrophobic/hydrophilic characteristics, have been demonstrated by researchers to be suitable candidates for wastewater technology [12,13]. These approches have been used in several studies to remove dangerous microbes, organic and inorganic contaminants, and poisonous heavy metal ions [14,15,16]. Therefore, the purpose of the current review is to outline the four types of nanomaterials’ potential contributions to the removal of these organic contaminants from wastewater. Additionally, this review summarizes the potential drawbacks of the techniques using nanomaterials as enhancements and highlights the difficulties that must be addressed in order to produce sustainable technologies in great detail.

## 2. Nanoreductive Processes

Nanomaterials can greatly improve water treatment and pollution control due to their exceptional adsorption capabilities, exceptional chemical characteristics, excellent mechanica properties, affordability, and power efficiency [17]. These compounds function as adsorbents and are distinguished by their clearly defined and controllable structures, as well as their suitable porosity and size [18]. NPs are used in wastewater to filter out other 1–100 nm particles, including viruses and organic material [19]. Unlike their larger cousins, which lack the same outstanding properties due to their small size and lack of structural and chemical features, NPs have impressive properties [20]. NPs frequently have a few hundred atoms in them [21]. The suitability of NPs for a given application is determined by a number of qualities, including physical, chemical, electrical, magnetic, and optical qualities, among others [22]. Other important chemical and physical properties exist in addition to the number of dimensions, form, chemistry, composition, surface size and arrangement, morphology, and hardness. NPs exhibit electrical properties based on their chemical composition in addition to their surface area, size, and chemical composition [9].

**Figure 2:**
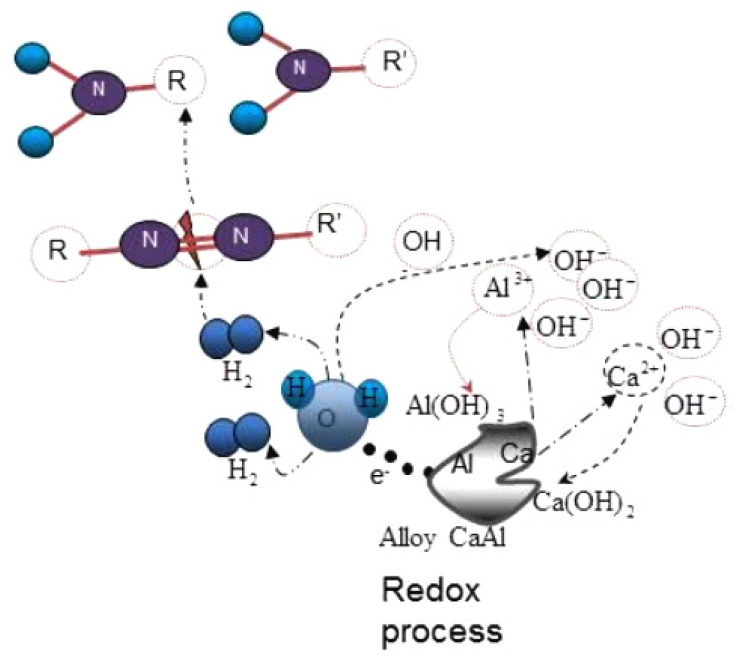
Schema of the proposed degradation mechanism of RB5 using Ca_65_Al_35_ compound. Reprinted/adapted with permission from Ref. [23]. 2017, Elsevier.

For industrial-level dye removal, reductive degradation is a rapid and inexpensive procedure that is simple to use. The following particles, including nanoparticles, have been used to date to remove azo dyes. A schematic representation of the Ca–Al system’s role in Mbarek et al.’s hypothesized degradation mechanism for RB5 is shown in Figure 2 [23]. Owing to its tiny particle size, nanoscale zerovalent iron (nZVI) quickly agglomerates to form necklace-like arrangements, according to experimental research [24,25]. The high reactivity of nZVI systems can only be attained when the wastewater is in an acidic condition (pH below 5.0), according to Xiong et al. [26].

A laboratory-scale slurry reactor system was used by Bigg et al. to explore the kinetics of reductive degradation of two distinct azo dyes in aqueous solution. The reaction kinetics for the reductive degradation of both dyes exhibited pseudo first-order behavior. At mixing speeds of 2000 revolutions per minute, the average rate constant for the reactions was 0.735 min^−1^ and 0.694 min^−1^ for acid orange II and acid blue 113, respectively. The apparent rate constant (k) was reduced with the initial dissolved oxygen content and pH, but the mixing speed and iron content both led to increases in this value. Both acid orange II and acid blue 113 exhibited a linear increase with iron concentration, with correlation coefficients of 3 × 10^−5^ mg L^−1^. For both dyes, the rate constant rose linearly with the inverse square root of the mixing speed, directly proportional to the rise in reaction rate as the thickness of the boundary layer decreased. Acid orange II’s half-lifetime correlated closely with the values given in the literature. A qualitative description of the impact of mass transfer on the reaction rate is provided by the mass transfer theory, and a quantitative one is provided by the empirical Sherwood number and the thickness of the stationary boundary layer that attaches to the iron particle. In terms of the initial dissolved oxygen concentration in the aqueous phase, the impact of surface passivation is quantitatively proved [27].

Mixing nZVI with a noble metal is a well-documented method used to increase the material’s chemical reactivity (Pd, Pt, Ag, Ni, etc.). A lot of experimental works using bimetallic nZVI for pollutant remediation have been conducted recently, including Fe/Pd [28,29,30,31,32], Fe/Pt [32], Fe/Ag [33], and Fe/Ni [29,34,35,36,37]. Fe^0^ is thought to act as an anode in these electrochemical couples, becoming sacrificially oxidized to galvanically preserve the noble metal. Particle performance in experiments has varied, with Fe–Pd generally outperforming the other combinations. It is thought that the noble metal directly transfers electrons to the sorbed pollutants at the bimetallic nZVI surface, or that hydrogen produced by the oxidation of Fe^0^ reacts with the contaminants. Most likely, there is hydrogen in the form of a dissolved gas, some of which is adsorbed to the surface of the particles, and an undetermined portion that may be present as an active metal hydride that underwent diatomic dissociation and reacted with the exposed noble metal [29].

High entropy alloys (HEAs) are recently developed materials in the field of metallic materials research [38]. Many desirable characteristics of HEAs have been discovered in recent scientific and technological research, including their mechanical properties [39,40,41], strong temper-softening resistance [39,40], good thermal stability [42], and great corrosion resistance [43,44]. Due to their fast decolorization, environmental protection, low cost, and low efficiency [45,46], high entropy alloys have the potential to be used in wastewater treatment. At this time, it has been reported that zerovalent iron [47,48], Mn–Al binary alloys [49], and Al-based metallic glass alloys [50] show considerable decolorization properties for azo dyes. Additionally, it has been shown that a number of elements, including Fe, Cr, and Mn, which all have BCC crystal structures, and Al, Ti, Zn, and Co, exhibit significant activity. Even though aluminum has an FCC crystal structure, its high activity allows it to operate as a component of an alloy with a high entropy, which leads to the creation of the BCC phase [51].

Wu et al. reported on the decolorization of the azo dye direct blue 6 (DB6) using high entropy alloys (HEAs): the ball-milled (BM) AlCrFeMn and AlCrFeMnM (M = Mg, Ti, Ni). These alloys have significant azo dye decolorization properties and are in line with pseudo first-order exponential decay kinetics. AlCrFeMn had a superior performance to BM MgZn-based glassy powders, decolorizing DB6 three times more rapidly. AlCrFeMnMg and AlCrFeMnTi had a reaction activity that was approximately 2 and 1.2 times higher than AlCrFeMn, respectively. In the decolorization of the azo dye DB6, BM AlCrFeMn and AlCrFeMnMg operated excellently, and their reaction activities were three and seven times faster than BM MgZn-based metallic glass, respectively. It is more difficult to decolorize materials with increasing dye concentrations. In comparison to neutral solutions, AlCrFeMnMg reacted around 37.5 and 16.6 times more rapidly in alkaline and acidic azo dye solutions, respectively. In the application of azo dye decolorization and degradation, HEAs have good research value and do not contribute to secondary pollution [39].

According to Sha et al., hollow Co nanoparticles were produced using the galvanic replacement process, showed rapid catalytic activity during the degradation of methyl orange, and easily converted methyl orange into amine compounds. The azo dye degradation efficiency reached up to 99% in 4 min, and the degradation constant rate was up to 2.444 min^−1^, with an initial methyl orange concentration of 100 mg/L (pH = 2.5) and 0.5 g/L of Co nanoparticles. The pH was a major factor in the dye degradation rates, and acidic environments were ideal. The rate at which methyl orange degrades is increased by the hollow nanoparticles’ significantly higher surface area for high concentration organic chemical adsorption and surface reactionscompared to solid nanoparticles [52].

According to works by Mbarek et al. and Abolighasembadi et al. [49,53,54], mechanically alloyed powders of manganese–aluminum (Mn85Al15) and calcium–aluminum (Ca65Al35) [23] demonstrated good efficiency and rapid response rates as decolorization agents for azo dye aqueous solutions. First, the effectiveness of powders produced using various manufacturing processes was compared in order to study the impact of the crystalline phase and microstructure. Second, the effectiveness of decolorization was examined using several dyes and actual textile wastewater samples. Although significant adsorption on the metallic surface was noted for several colorants, an analysis of the treated water and particle samples revealed that the predominant reaction mechanism included the degradation of azo dye molecules. Finally, it was determined whether the particles could be reused and whether the treatments had successfully reduced the toxicity. Manganese–aluminum powders have a variety of advantages over other highly efficient decolorizing metallic compounds, including easy production and application processes, high efficacy, and the use of environmentally favorable metallic elements. Additionally, Mbarek et al. investigated how adding Fe and Co affected the ability of mechanically alloyed Mn–Al powders to degrade azo dyes. The Mn–Al–Fe powder had remarkable degrading efficiency, and it was demonstrated that the reaction’s kinetics were more rapid than those of Mn–Al-based alloys containing 10% Fe and 10% Co. The valence electron configuration was related to the high efficiency of the Mn–Al–Fe powder. Due to the formation of a localized adsorption bond with the adsorbate molecule, this favors a higher concentration of reactive (hole) sites in the d-band for iron compared to cobalt, favoring a higher adsorption capacity. Moreover, in order to facilitate the creation of hydrogen from water, the zerovalent metals iron (Fe^0^) and cobalt (Co^0^) act as reactive agents. The azo dye solution’s decolorization follows first-order kinetics principles as well. As a result, the high effectiveness of Mn–Al-based alloys observed in decolorization treatments of dyed wastewater, as noted in prior studies, can be adjusted and augmented by moderate alloying with additional transition metals [55]. The ball-milled Ca65Al35 powder also demonstrated remarkable degrading efficiency and high response rates, removing more than 90% of a 40 mg L1 dye solution in less than a minute. These materials may be employed as a low-cost, high-performance decolorizing approach for textile wastewater pretreatments due to their huge surface area, high efficiency, and chemical activity. The reducing capability of Ca serves as the foundation for a mechanism in an aqueous basic media [23]. Table 1 shows some nanoreactives applied in the removal of organic pollutants.

**Figure 3:**
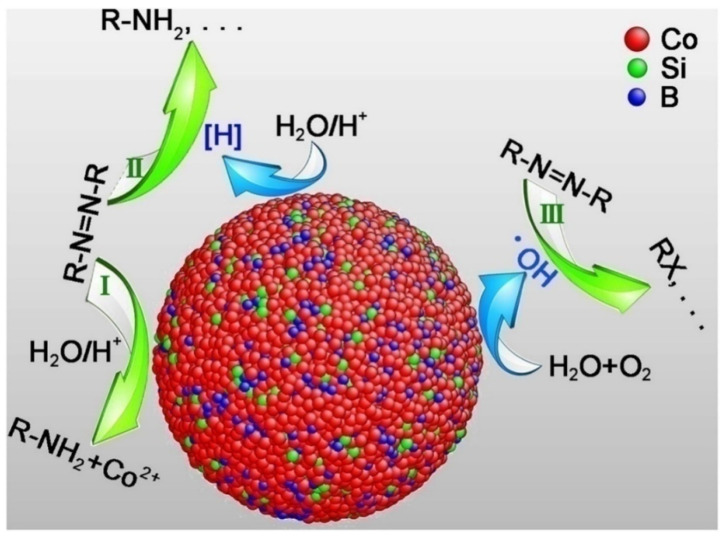
Illustration drawn by Qin et al. of the major reactions occurring in the amorphous alloys and the degradation mechanisms [66].

The use of amorphous alloys is a new approach that has been studied extensively by several research groups. Due to their reduced mass loss, these alloys often exhibit superior corrosion resistance than their crystalline equivalents [64,65]. The amorphous structure of Co-based MG powders is responsible for the rapid degradation of azo dyes by these materials. The potential degradation reactions and mechanisms are depicted in Figure 3 [66].

In comparison with traditional metals, many amorphous alloys have been produced to increase the degradation efficiency of azo dyes in wastewater. Table 2 details the effectiveness and experimental parameters for azo dye removal using amorphous alloys, including the pH solution, initial dye concentration, and alloy dosage. It can be said that amorphous alloys based on Fe, Mg, Co, Al, and Mn are effective at removing various azo dyes from wastewater.

## 3. Nanophotocatalysis

### 3.1. Nanocatalysis

#### 3.1.1. Nanomaterials as Photocatalysts

Nanocatalysts are attracting a lot of interest as wastewater treatment materials (especially those nanomaterials made of inorganic substances such as semiconductors and metal oxides). For the purpose of the analysis of oxidation of organic pollutants [72] and antimicrobial effects [73], a variety of nanocatalysts are used in wastewater treatment, including photocatalysts [74,75], electrocatalysts [74], heterojunction photocatalytic materials [76], and Fenton-based catalysts [77] (Table 3).

Due to their extensive and efficient photocatalytic activity for diverse contaminants, nanoparticle photocatalytic reactions, which are based on the interaction of light energy with metallic nanoparticles, are of enormous importance. Usually made of semiconductor metals, these photocatalysts can decompose a variety of persistent organic contaminants found in wastewater, including dyes, detergents, insecticides, and volatile organic compounds [78]. Additionally, semiconductor nanocatalysts are highly efficient at degrading both halogenated and nonhalogenated organic chemicals, as well as certain medications, personal care items, and heavy metals [79]. Semiconductor nanomaterials work well, even at low concentrations, and require relatively benign operating conditions. In the same cotext, Baaloudj et al. [80] deduced that the photocatalytic performance of catalysts varies, which was mostly caused by the experimental conditions and, more significantly, by the bandgap, which was crucial to the photocatalytic activity. Depending on the catalyst’s color and the components that it is comprised of, the bandgap varies from one catalyst to another. This distinction can be explained by the process in which a semiconductor can be quickly stimulated by light illumination, which absorbs photons with an energy greater than or equal to that of its bandgap energy, and can then be excited to generate electron–hole pairs (e^−^ and h^+^) [81]. After that, O_2_ on the surface of the semiconductor will react with the e^−^ on the conduction band to form O_2_^−^* radicals through a reduction process. The hydroperoxyl radical H_2_O* is then produced by protonation [82]. The trapped electrons, subsequently, combine with these radicals to form hydrogen peroxide H_2_O_2_ and hydroxide radicals OH*. The photogenerated valence band holes can react with either water H_2_O or the hydroxyl ions OH^−^ adsorbed on the catalyst surface to produce OH* radicals, which are powerful oxidants and the primary active oxygen species in the photocatalytic destruction of organic contaminants [83]. Accordingly, it is possible that h^+^ makes up a portion of the OH radicals, aiding in the breakdown of organic contaminants [84]. Baaloudj et al. [80] have, therefore, characterized the process by which organic pollutants are broken down to produce electron–hole pairs and active radicle species as follows:

Catalyst + hν → Catalyst* + e^−^ + h^+^
H_2_O + h^+^ → OH* + H^+^
O_2_ + e^−^ → O_2_^−^*
e^−^ + 2H^+^ + H_2_O* → H_2_O_2_
H_2_O_2_ + e^−^ → OH_(e−)_ + OH*
Organic pollutants + O_2_^−^* + OH* → CO_2_ + H_2_O + other small products

**Figure 4:**
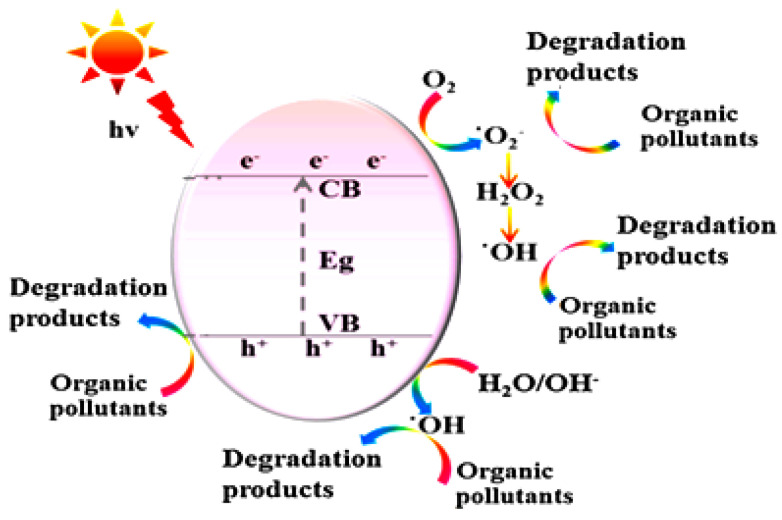
General mechanism of photocatalysis, depicting the generation of holes (h^+^) and electrons (e^−^) [80].

Figure 4 shows a schematic diagram for the proposed mechanism of photocatalysis, depicting the generation of holes (h^+^) and electrons (e^−^).

Several decades ago, the idea of utilizing TiO_2_ nanoparticles for photocatalytic dye degradation was proposed [85]. Silver nanoparticles produced by green synthesis have also been applied as photocatalysts to process dyes and other organic compounds [86]. Nanotechnology was not widely used for wastewater treatment at the time, but it is now because of evidence of its great effectiveness [87]. Gold nanoparticles (GNP) with a diameter of 43 nm were employed by Singh et al. to study the photocatalytic degradation of methylene blue (MB). The calculated catalytic degradation at 2 and 3 min was 78.97% and 96.26%, respectively. The fact that GNP’s redox potential was decreased to a negative value at the nanoscale explains its catalytic activity [88]. Haque et al. have concentrated on photocatalytic activity, employing La- and Mo-doped TiO_2_ hybrid carbon spheres with substantial adsorption capacities, particularly for organic chemicals, such as azo dyes, in light of these characteristics. In 120 min, a 2.0% La-doped TiO_2_ and a 1.5% Mo-doped TiO_2_ decomposed 94% and 44% of Acid Green 25, respectively. In this case, organics and dyes were changed into harmless materials by La- and Mo-doped TiO_2_. When compared to other dopant concentrations, the photocatalytic activity of TiO_2_ with dopant concentrations of 2.0% (La) and 1.5% (Mo) demonstrated the highest effectiveness for the degradation of azo dyes [89].

#### 3.1.2. Nanomaterials as Electrocatalysts

In recent decades, electrocatalysis has undergone significant progress. Numerous fascinating themes in the field of catalysis have arisen as a result of the distinctive characteristics of numerous nanostructures of electrocatalytic materials and their surfaces. Electrocatalysis, a crucial area of catalysis, is a crucial type of catalytic reaction that can transform and store energy through processes involving the transport of electrons. However, because of the extremely complex reaction network, the wide range of reaction selectivity, and the perplexing reaction processes, studying electrocatalysis is extremely difficult. Rare-earth (Gd^3+^, Nd^3+^, and Sm^3+^)-doped cerium oxide has been successfully employed in textile color removal and the decomposition of azo dye RO 107, demonstrating a significant effect on the electrocatalytic activity, according to Rajkumar et al.’s study. The electro-oxidation and electrocatalytic oxidation procedures enable the degradation of high concentration and highly chromoselective dye solutions. The proposed electrochemical degradation process is a successful method of decolorization, according to UV-Vis and FTIR spectral analyses. According to mineralization experiments on RO 107, electrocatalytic oxidation utilizing ceria oxides doped with Gd^3+^, Nd^3+^, and Sm^3+^ increased TOC removal values from 32 to 35.7% after 20 min. The azo bond of the dye structure was the first potentially damaged when the azo bond was attacked, which resulted in the decolorization of the dye. This intermediate was discovered with the GC–MS technique. The intermediates continued to be decomposed into carbon dioxide and water with the aid of the hydroxyl radical and other radicals, which caused the dye solution to become mineralized. When combined with electro-oxidation, cerium-doped Gd^3+^, Nd^3+^, and Sm^3+^ oxides are excellent at removing contaminants from textile dye effluent rapidly. Further research on this method could be performed to find alternative methods for treating wastewater. The findings showed that the cocatalysts for electrocatalytic oxidation processes are Ce_0.8_Gd_0.2_O_2_, Ce_0.8_Nd_0.2_O_2_, and Ce_0.8_Sm_0.2_O_2_. The swift suppression of the electrocharge carriers by the catalyst was the primary electrochemical process responsible for the increased rate. An electron acceptor’s favorable function is the production of additional radicals, which effectively degrade the contaminants through the radical chain branching mechanism. To test this at the industrial scale and with other types of organic effluent, more research is required [90].

Using an electrocatalytic vanadium-doped TiO_2_ nanocatalyst, Chang et al. examined the degradation of acid red 27 (AR 27). The results showed that AR 27 may be successfully degraded by nano-V/TiO_2_ electrodes; the greatest color and total organic carbon (TOC) removal efficiencies reached 99% and 76%, respectively, under 0.10 VT (molar ratio of vanadium to titanium) conditions. A high specific surface area nano-V/TiO_2_ electrode aided in electrocatalytic degradation. This electrocatalytic device performed best at a current density of 25 mA cm^2^, and as the current density grew, more oxygen was produced. In this electrocatalytic system, the electrical consumption of the nano-V/TiO_2_ electrode and pure-TiO_2_ electrode was approximately 0.11 kWh L^−1^ and 0.02 kWh L^−1^, respectively. As a result, it can be concluded that the nano-V/TiO_2_ electrode had both high degradation and energy-saving qualities. The nano-V/TiO_2_ electrode also demonstrated its potential for repeated use [91].

#### 3.1.3. Heterojunction Photocatalytic Material

**Figure 5:**
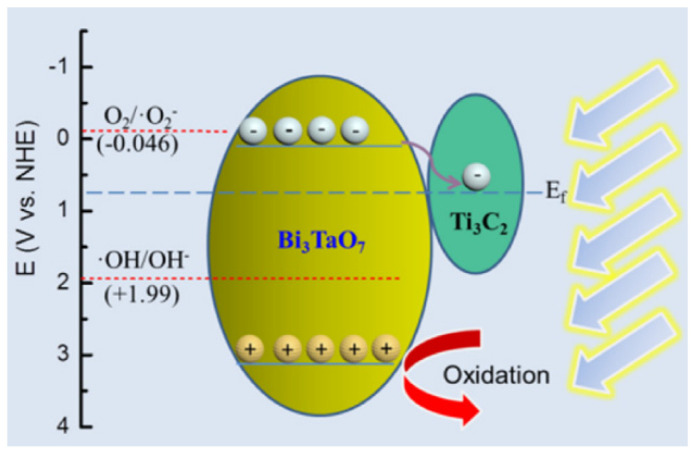
The proposed mechanism for MB removal with BTC-10 under visible light irradiation [107].

The heterojunction photocatalytic material, among the different proposed technologies, has the most potential, since it directly uses solar energy for the creation of valuable chemical fuels (hydrogen, hydrocarbon fuels, etc.) as well as the degradation of hazardous pollutants [92,93,94,95,96,97]. Numerous semiconductors have been researched and produced since Honda and Fujishima’s research on photocatalysis in 1972 [98,99,100,101]. The spatial separation of photogenerated electron–hole pairs was shown to enable the appropriately constructed heterojunction photocatalysts to possess enhanced photocatalytic activity. BiVO_4_/CeO_2_ type-II heterojunction photocatalysts for the photocatalytic degradation of methylene blue (MB), methyl orange (MO), and a combination of MB and MO were synthesized hydrothermally, according to Wetchakun et al. [102]. It was discovered that the pH values of 4.56 for BiVO_4_ and 7.33 for CeO_2_ corresponded to differing isoelectric points. It has been demonstrated that the difference in isoelectric points between these two semiconductors is advantageous for simultaneously adsorbing cationic and anionic dyes. Particularly, during degrading events, the BiVO_4_ and CeO_2_ can each preferentially adsorb cationic MB and anionic MO, respectively. Due to the electrostatic repulsion between the surface charges of the photocatalysts and the charges of the dye molecules, the BiVO_4_/CeO_2_ composite had stronger photocatalytic-degradation activity toward the mixture of MB and MO than the individual BiVO_4_ or CeO_2_ photocatalysts. The improved electron–hole separation efficiency and potent electrostatic interaction between the composite and the dye molecules were credited for the exceptional activity of the composite photocatalyst. According to this work, the appropriate coupling of two different semiconductors could both increase the effectiveness of electron–hole separation and provide photocatalysts with good adsorption toward both anionic and cationic dyes [76]. Hu et al.’s study of the linked semiconductor Cu_2_O/CeO_2_ photocatalyst’s catalytic activity in the presence of visible light revealed that this heterojunction semiconductor photocatalyst had 20% more photocatalytic degradation of acid orange 7 (AO_7_) than pure CeO_2_ [103]. The development of p–n junctions was responsible for the highest photoactivity. Li and Yan [104] examined the photocatalytic degradation of Rhodamine B over a Bi_2_O_3_/CeO_2_ catalyst when it was exposed to visible light. The rhodamine B substrate was totally destroyed within 8 h of irradiation, according to their findings, which showed that Bi_2_O_3_/CeO_2_ in a 9:1 molar ratio gave the greatest photodegradation activity. The improved charge carrier lifetime that was attained by using a composite photocatalyst was connected to the improvement in photocatalytic efficiency. The photocatalytic degradation of rhodamine B over ZnO/CeO_2_ composite nanofibers was studied by Li et al. [105]. They found that the composite photocatalyst was able to completely degrade the dye substrate within 3 h, while only 17.4% and 82.3% degradation was acquired in the case of pure CeO_2_ and pure ZnO, respectively. Li et al. studied the photocatalytic degradation of methylene blue (MB) using Bi_3_TaO_7_/Ti_3_C_2_ heterojunctions, and they deduced a removal efficiency of approximately 99% after 2 h [106]. They reported that synergistic effects between Bi_3_TaO_7_ and Ti_3_C_2_ improved the photocatalytic performance by enhancing electron–hole pair separation, electronic transmission efficiency, and interfacial charge transfer. They concluded that Ti_3_C_2_ serves as an “electronic highway”, isolating the photoelectron–hole pairs and enhancing photocatalytic activity. According to their hypothesized photocatalytic mechanism, which is depicted in Figure 5, photogenerated electrons swiftly migrate on Bi_3_TaO_7_ and congregate on Ti_3_C_2_ nanosheets. The hydroxyl groups are oxidized by holes gathered on Bi_3_TaO_7_ to produce OH*, which is an essential oxidant for dye removal. MB can be oxidized immediately by h^+^ to produce the tiny non-toxic molecules H_2_O and CO_2_ at the same time.

Huang et al. [107] studied the photocatalytic degradation of a Bi_2_S_3_/Bi_2_O_3_/Bi_2_O_2_CO_3_ nanocomposite. They demonstrated that this manufactured nanocomposite had superior photocatalytic activity in the breakdown of organic contaminants when exposed to visible light. However, under visible light, these Bi_2_S_3_/Bi_2_O_3_/BOC catalysts could remove 99% of HCHO (500 ppm) in 100 min and were able to remove more than 99% of MO in just 60 min. They claimed that the greater light absorption and effective charge separation were responsible for the composite’s much better performance, and the mechanism behind this high photocatalytic activity showed that superoxide and holes, as opposed to hydroxyl radicals, dominate the photocatalytic process.

**Figure 6:**
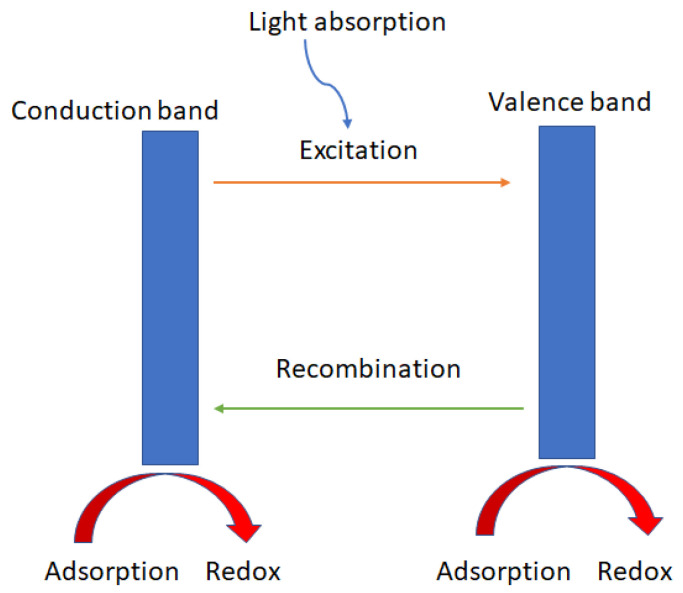
Schema of the typical photocatalytic processes on a heterojunction photocatalytic material.

According to Low et al., there are at least five basic steps in the photocatalytic process of a semiconductor: (i) the semiconductor’s ability to absorb light, (ii) the generation of photogenerated electron–hole pairs, (iii) their transport and recombination, (iv) the adsorption of reactants and the desorption of products, and (v) the activation of redox reactions on their surface (Figure 6) [76].

#### 3.1.4. Nanomaterial-Based Fenton Catalysis

Using the Fenton reaction to improve the effluent’s biocompatibility or transform the majority of the organic contaminants into low-molecular-mass carboxylic acids and even CO_2_ is one of the most economically advantageous ways to treat wastewater with low-to-medium levels of total organic carbon. By reducing H_2_O_2_ with Fe II, hydroxyl radicals that are extremely aggressive are produced in the Fenton reaction. As an alternative to Fe II, transition metal ions, such as Cu^+^ and Mn^2+^, can also aid in the process’s progress [108] There have been numerous reports of homogeneous catalysts being effective for the Fenton reaction [109]. The benefit of heterogeneous catalysts is that they make it simpler to separate material from effluent and eventually reuse it. As a result of this, heterogeneous catalysis are viewed as a natural development of homogeneous catalysis [110]. The creation of heterogeneous catalysts for the Fenton reaction has seen a rise in activity in recent years [108,111]. Recently, a review of the development of clays, silicas, and zeolites for heterogeneous Fenton catalysts was published [108]. Activated carbon has been used by numerous research groups either as a heterogeneous catalyst for the Fenton reaction or in other processes. Additionally, it is capable of supporting metals and metal oxides that have catalytic properties for the Fenton reaction (Table 3) [108]. Reviewing the usage of metal nanoparticles as heterogeneous catalysts for the production of OH radicals from H_2_O_2_ is also interesting, given the constant increase in the application of the Fenton reaction for the treatment of several industrial effluents. A new generation of nanoparticle-based heterogeneous catalysts has recently been created for the Fenton reaction. Due to the fact that they can have unique characteristics from the macroscopic or bulk forms of the same material, nanoparticles are important [108]. It is well known that some nanoparticles in catalysis have catalytic characteristics that are missing from bulk materials [112]. Additionally, the size, shape, surface structure, and bulk composition of nanocatalysts all have a significant impact on their activity and selectivity.

**Table 3:** List of nanophotocatalysts in the removal of organic pollutants.

Alloy	Route of Synthesis	Organic Pollutants	Source of Light	Pollutant Concentration	Catalyst Dose	pH	Time	Removal	Ref.
CuO nanosheets	Room temperature	Alura red Ac	UV	5 mg/L	5 mg	Neutral	6 min	96.65%	[113]
MFe_2_WO_6_ (M = Co,Ni, Cu,Zn)	Coprecipitation–oxidation method	Methyl red methyl orange Methylene blue bromo green	UV	10 mg/L	100 mg	Neutral	50 min 50 min 50 min 50 min	78% 92% 89% 93%	[114]
Cds/CuS	Hydrothermal method	Methyl orange	UV	10 mg/L	30 mg	Neutral	150 min	93%	[115]
α-Bi_4_V_2_O_11_	Combustion method	Rhodamine B	UV	5.106 mol/L	0.5 g/L	Neutral	6 min	100%	[116]
TiO_2_ graphene	Chemical Synthesis	Reactive black 5	UV	42 ppm	3 g	Neutral	40 min	96%	[117]
Al_2_O_3_-NP/SnO_2_	Sol-gel technique	Methyl orange	UV	20 mg/L	Electrode area 4.5 cm^2^	7	50 min	93.95%	[118]
CuO-Go/TiO_2_	Hydrother-mal method	2-chlorophenol	UV	50 mg/L	0.05 g/L	5	210 min	86%	[119]
CuO nanorods	Chemical synthesis	Reactive black 5	UV	20 µM	20 mg	Neutral	300 min	98%	[120]
Cu/Cu(OH)_2_	Coprecipitation	Rhodamine B	UV	100 ppm	20 mg/L	Neutral	120 min	99.99%	[121]
Copper nanoparticles	Hydrothermal method	Phenyl red	UV	10 mg/L	30 mg	Neutral	15 min	99.62%	[122]

## 4. Nanoadsorbents

**Figure 7:**
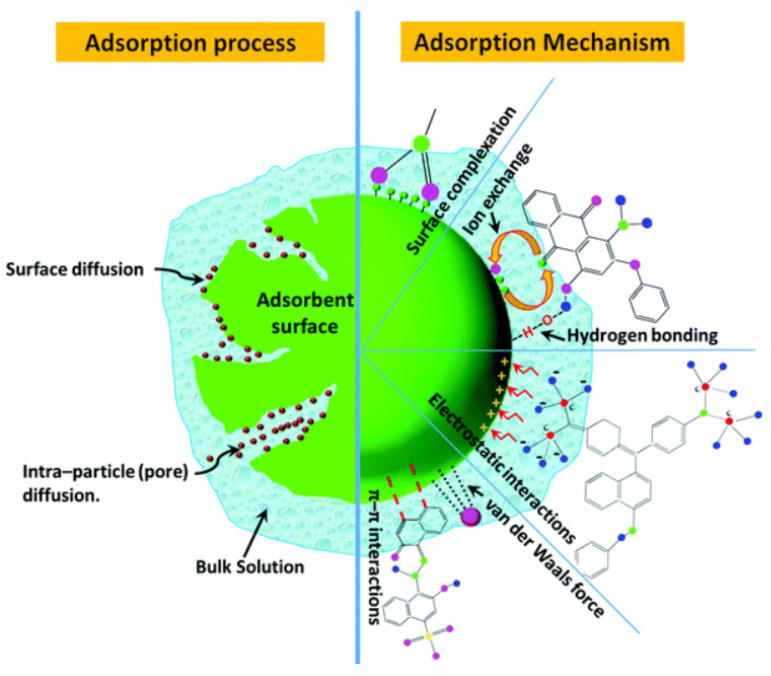
Adsorption processes and mechanisms for dye removal from bulk liquid [123].

As schematically depicted in Figure 7 [123], several adsorption methods are used to successfully adsorb dye from polluted waters onto the surface of an adsorbent. It should be mentioned that electrostatic attraction, π–π interactions, van der Waals forces, hydrogen bonds, acid–base reactions, and hydrophobic interactions are the key mechanisms controlling the adsorption of water contaminants on adsorbents. The features of nanoparticles that make them appropriate as nanoadsorbents for sequestration of any cleanup procedure are a high splitting coefficient, chemical and thermal stability in the solvent, chemical inertness, high porosity, being easy to remove from a solution after adsorption, sensitivite and selective towards the target pollutant, being easily regenerable and reusable numerous times, and easy and inexpensive to manafacture.

The primary intrinsic characteristics of nanoadsorbents, such as their fundamental functional groups and surface modification, are being studied in order to improve their capacity to remove hazardous pollutants throughout the process of wastewater treatment as shown in Table 4, Table 5, Table 6 and Table 7. By creating nanocomposites, such as silver/carbon, carbon/titanium oxide, etc., substantial efforts were also made to reduce toxicity. The use of nano-adsorbents in wastewater treatment is the most encouraging method due to their cost-effectiveness, biocompatibility, ease of commercialization, toxic-free method, biodegradability, use of less trained workers, selective separation, ease of recovery, and, most importantly, their high efficacy in removing pollutants.

The following characteristics, including size, shape, surface chemistry, aggregation ability, crystallinity, and chemical reactivity, among others, are crucial in determining how effectively a procedure removes contaminants from an aquatic environment [124].

### 4.1. Metal Oxide-Based Nanoadsorbents

The inorganic compounds known as metal oxide adsorbents have the distinctive qualities of an increased surface area, high solubility, and reduced production of secondary contaminants. These metal oxide nanoadsorbents can mediate electrostatic interactions due to their charged surfaces, which helps the solute transfer process.

In order to overcome the impacts of fragility, aggregation, and a pressure drop, among other things, a large number of research works on the synthesis of stable nanomaterials and engineering them with the specified functional molecules have been reported. The synthesis of stable nanoceria with amine functionalization was previously reported, and the adsorption procedures used against anionic azo dyes, such as acid yellow 36 and acid yellow 17, were shown to be successful [125,126].

The use of metal oxides to sequester water contaminants has been the subject of numerous studies in recent years (Table 4) [125,127,128]. Their contribution to the environment comes in the form of nanoscale CeO_2_ or nanoceria, which functions as a photocatalyst for the decomposition of dyes. Their suitability for the absorption of heavy metal ions is determined by the defined surface features of nanoceria and acceptable electrical charge values [129]. Rajarathinam et al. focused on the synthesis and surface functionalization of nanoceria, and explored their adsorption capability for the removal of azo dye Fenalan Yellow G (FYG) taking these properties into consideration. With an adsorbent dosage of 0.1 g for a dye concentration of 10 mg/L of FYG, the maximum removal of 93.62% was seen after 210 min at a pH of 2.0. According to these results, surface-functionalized nanoceria (sf-gNC) can be used as a substitute material for traditional adsorbents in dye-removal procedures [126].

Studies in this topic have been conducted on how heavy metal ions or colors that are pollutants in wastewater degrade. In order to remove malachite green oxalate (MGO) and hexavalent chromium (Cr) from an aqueous solution, Kumar et al. effectively generated metal oxide nanoparticles, such as ZnO and SnO_2_, using a precipitation technique, with specific surface areas of 15.75 and 24.48 m^2^/g, respectively. ZnO and SnO_2_ had 95% and 92% efficiency in decolorizing MGO, respectively. Similarly, Cr adsorbs to ZnO and SnO_2_, and they removed 95% and 87% of Cr, respectively [130].

**Table 4:** Azo dye degradation with oxide-based nanoparticles.

Alloy Name	Organic Pollutants	Pollutant Concentration	Nanoadsorbent Dosage	pH	Time	Degradation Efficiency	Ref.
TiO_2_/MgO	Methyl orange alizarin red S	5 ppm	0.5 g	Neutral	90 min	83.2% 43.8%	[131]
CeO_2_	Methylene blue	30 mg/L	200 ppm	Neutral	60 min	98%	[132]
ZnO/CuO	Congo red	50 ppm	50 mg/L	5.6	30 min	93%	[133]
CuO/γ-Al_2_O_3_	Brilliant red X-3B	0.30 g/L	5.50 g/L	8	2.50 h	90.72%	[134]
Iron oxide nanoparticles	Metanil yellow Orange II	20 ppm	8 mg/L	Neutral	7 h	95% 67%	[135]
MnO_2_/SnO_2_	Calcon dye	15 mg/L	Film	3	24 h	93.5%	[136]
α-MnO_2_/TiO_2_	Coomassie brilliant blue R-250	13 mg/L	1 g/L	3	30 min	98.35%	[137]
Bi_2_WO_6_/ MnO_2_	Methylene blue	10 mg/L	100 mg/L	7	100 min	100%	[138]

### 4.2. Carbon-Based Nanoadsorbents

Due to the distinctive atomic structure of the carbon atom, carbon materials exhibit a variety of structures and special qualities [139,140]. Carbon materials are classified as zero-dimensional nanomaterials (Buckminster fullerenes and carbon dots), one-dimensional nanomaterials (carbon nanotubes and carbon nanofibers), two-dimensional nanomaterials (graphene), and three-dimensional nanomaterials (carbon sponges) based on their shape, size, and dimensionality [141].

Carbon nanotubes (CNTs) can be found in two-dimensional graphene sheets or three-dimensional nanotubes. They can be divided into two categories based on how many layers or sheets are folded: single-walled CNTs (SWCNTs) and multi-walled CNTs (MWCNTs). SWCNTs play a significant role in clean-up strategies due to their increased surface area, numerous adsorption sites, and other characteristics. The adsorption of a solute on the surface of the adsorbent can be mediated by conventional hydrophobic interactions. As a result, these CNTs are among the adsorbents that have received the most attention in recent years for their capacity to remove a variety of heavy metal ions and organic dyes from wastewater [142].

The maximum adsorption capacity of a given organic pollutant on CNTs was determined by the CNT surface area, surface functional groups on CNTs, the pores in CNT aggregates, surface curvature, and defects of CNT monomers, according to Yang and Xing’s review of the aqueous adsorption of organic pollutants using various carbon nanomaterials [143]. The ability of organic pollutants to adhere to CNTs depends in part on the structures and characteristics of the contaminants. The diverse surfaces of CNTs interact in a variety of ways with distinct molecule configurations. To thoroughly analyze the adsorptive interactions between CNTs and organic pollutants, Chen et al. studied the adsorption of contaminants with different physical-chemical properties to three different types of CNTs. They discovered that while the adsorption affinity did not significantly correlate with hydrophobicity, it did rise in the following order: nonpolar aliphatic, nonpolar aromatic, nitroaromatic, and with the number of nitrofunctional groups within the nitroaromatic group [144]. As a result, the type, quantity, and position of functional groups in organic molecules determine how organic pollutant functional groups affect the adsorption of organic pollutants. The order of the substituted groups at a given position following aniline and phenol’s affinity for adsorption on CNTs is as follows: nitro group > chloride group > methyl group [145]. Table 5 provides details of the azo dye degradation with some carbon nanotube materials.

**Table 5:** Azo dye degradation with carbon nanotube materials.

Alloy Name	Organic Pollutants	Pollutant Concentration	Nano-Sorbent Dosage	pH	Time	Degradation Efficiency	Ref.
Multiwall carbon nanotubes (MWCNTs)	Methyl blue	25 mg/L	10 mg/L	6	10 min	99%	[146]
Carbon nanotubes grown on carbon fiber	Methylene blue	5 mg/L	Electrode	1.83	180 min	100%	[147]
ZnO/NiO with multiwall carbon nanotubes	Methyl orange	50 ppm	3% NiO:92% ZnO:5% CNTS	7	360 min	71%	[148]
Multiwall carbon nanotubes	Reactive Black 5	15 mg/L	3 g/L	7	60 min	100%	[149]
Single-wall carbon nanotube Ru nanoparticle	Congo red	0.05 mM	0.3 mg	5	4 min	97.5%	[150]
Single-wall carbon nanotubes	Reactive yellow dye 15	50 mg/L	0.2 g/L	3	5 min	179.9mg/g	[151]
Carbon nanotubes/alumina	Congo red	10 mg/L	15 mg	2	90 min	96.4%	[152]

The adsorption capacities of graphene were much higher than those of other adsorbents under similar conditions, making it a promising adsorbent for the removal of heavy metal ions such as Au(III), Pt(IV) [153], Pb(II) [154], Cu(II) [155], Zn(II) [156], Cd(II) [157], and Co(II) [158]. Graphene is one of the most surprising modern carbon allotropes with distinct properties. The most well-known technique of chemical synthesis is the Hummers method [159], which involves oxidizing graphite to create the two-dimensional oxide form of the compound. With a focus on catalytic and degrading activities, the effectiveness of testing graphene oxide (GO) for the elimination of different dyes was thoroughly examined (Table 6) [160]. TiO_2_–graphene composites, among a variety of other graphene composites, are frequently employed for the photodegradation of dyes. At the same time, the photocatalytic degradation of dyes, and properties of several metals and metal oxide composites were investigated. The most often studied dye among them for degradation was methylene blue, followed by rhodamine B [160]. Different graphene materials have been used to study dye absorption as well as photocatalytic degradation. Rhodamine B (RB), malachite green (MG), and acriflavine (AF), which are carcinogenic dyes, were applied to highly porous, lightweight graphene oxide foams and high removal capacities were observed of 446, 321 and 228 mg/g, respectively [161]. A simple and affordable lyophilization procedure was used to create the specific 3D GO. Due to the 3D architecture, which is one advantage in terms of its practical use, the foam might be used directly without any preparation, such as in ultrasonication. The excellent antibacterial activity these foams have simultaneously shown against *Escherichia coli* bacteria in aqueous and nutrient growth conditions further suggests their potential for use in water treatment [161].

**Table 6:** Azo dye degradation with graphene oxide materials.

Alloy Name	Route of Synthesis	Organic Pollutants	Pollutant Concentration	Nanoadsorbent Dosage	pH	Time	Adsorption Capacity	Ref.
Graphene oxide	Modified Hummers method	Acid orange 8 (AO8) and direct red 23 (DR23)	50 mg/L	40 mg	7	60 min	AO8 = 25 mg/g and DR23 = 14 mg/g	[162]
Graphene oxide and magnetic chitosan	Modified Hummers method	Methyl blue (MB)	200 mg/L	0.015 g	5.3	60 min	95.16 mg/L	[163]
Graphene oxide-supported manganese oxide	Modified Hummers method	Reactive black 5	60 mg/L	0.01 g	3	24 h	87 mg /L	[164]

### 4.3. Silica-Based Nanoadsorbents

Sand-like silica is one of the Earth’s crust’s most abundant components. Owing to its special qualities as a lightweight material, silica is a necessity in the production of electrical and communication devices. Silica is utilized in traditional chromatographic methods to separate the desired solute from a complex mixture. One of the most popular uses for silica is the adsorption of pollutants; it has been widely used to remove colors, heavy metals, and other contaminants from drinking water [165]. As a result, numerous research studies on the synthesis of silica have been documented. The ability of a synthesized silica nanoparticle (SSN) to remove dye from single and multicomponent (ternary) systems was reported by Mahmoodi et al. Their findings indicated that the SSN, an environmentally benign adsorbent with a high capacity for cationic dye adsorption, would be a good substitute to remove dyes from multicomponent systems (as shown in Table 7) [166].

**Table 7:** Azo dye degradation with silica-based nanoadsorbents.

Alloy Name	Organic Pollutants	Pollutant Concentration	Nanoadsorbent Dosage	pH	Time	Degradation Efficiency	Ref.
Nanosilica particles	Methyl orange	10 mg/L	10g/3L	2.5	30 min	100%	[167]
Silica nanoparticles	Methyl red dye	0.05 mM	10g/L	7	120 min	95%	[168]
Mesoporous silica nanomaterial	Rhodamine B	20 mg/L	1g/L	5.8	120 min	98.92%	[169]
Mesoporous silica modified with L-arginine	Crystal violet	100 ppm	10 mg	11	30 min	100%	[170]
Porous silicon supporte porous ruthenium nanoparticle system	Condo red	1 mM	40 µl	5	60 min	96%	[171]
Titania-coated silica nanocomposite	Safranin-O dye	17.61 mg/L	89.80 mg/g	6.2	60 min	93.29%	[172]

## 5. Nanomembranes

**Figure 8:**
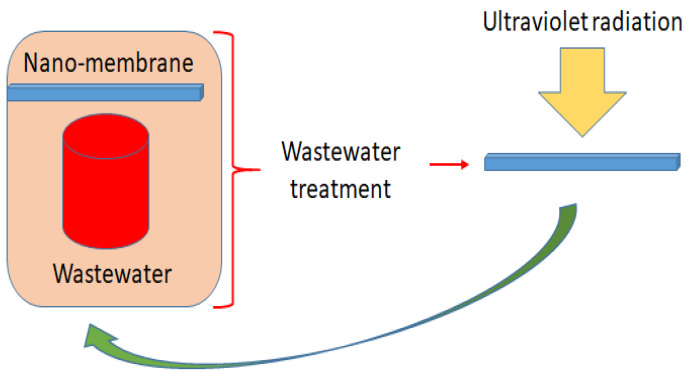
Schema of the nanomembrane procedure for wastewater treatment.

Using size exclusion and solution diffusion, nanomembranes, a special type of membrane made of various nanofibers, have been used to remove pollutants based on viruses, inorganic ions, and organic and inorganic nanoparticles from water resources. This method facilitates extremely high elimination rates with condensed fouling propensities, and it also serves as a pretreatment step for reverse osmosis [173]. Numerous studies on membrane nanotechnology have been published in an effort to create multifunctional membranes employing various nanomaterials in various polymer-based membranes [174]. Reverse osmosis, nanofiltration, and other water-treatment methods have used water-porous membranes. A porous support with a composite layer is present in the membrane. The significant composite layer is often composed of a carbon-based material (graphene oxide/CNT) spread in a polymer matrix. As depicted in Figure 8, this results in considerable and prospective advancements in water transport and fouling resistance.

The membranes are commercially available and appropriate for a wide range of uses. However, the effort to create new water resources from sewage calls for membranes with higher productivity and a lower cost related to fouling resistance. Organic substances in water interact with hydrophobic membranes to create membrane fouling. The accumulation of particles on the membrane’s surface or inside its pores is the cause of fouling. Almost all membrane processes experience membrane fouling, which is often brought on by precipitation and particle or molecule deposition on the membrane’s surface or in its pores [175]. Increased membrane separation resistances, decreased productivity, and/or altered membrane selectivity are the effects of membrane fouling [176]. Due to fluctuating product quality and poor recovery, this has an impact on the separation factor for the targeted species in the feed. Pore blockage and solute aggregation, which results in a cake development or a gel layer on the membrane surface, in addition to adsorption, which is exacerbated by concentration polarization and convective forces to and through the membrane, are all typical components of the fouling process. Membrane qualities, such as the material from which the membrane is formed, and feed solution properties, such as composition, concentration, pH, and ionic conditions, are the two main groups of factors that affect membrane fouling. In order to remove suspended solids and condition the feed and membrane surface to reduce the tendency for membrane fouling, pretreatments of feed that have an impact on the feed’s properties in membrane systems are crucial.

A high specific surface area and high porosity with small pores are two distinctive characteristics of electrospun nanofiber membranes. The development of an efficient technology for processing wastewater dyes has been the subject of numerous studies. A variety of techniques have been developed to remove synthetic colors from water and wastewater to reduce their environmental impact. Adsorption on inorganic or organic matrices, color removal via photocatalysis, oxidation, microbiological or enzymatic breakdown, etc., are some of the technologies used [177]. One of the most efficient and financially viable methods for removing textile colours from wastewater is adsorption [178].

The surface area and structure of an adsorbent are its most crucial characteristics. Additionally, the adsorbent surface’s polarity and chemical make-up may affect the attractive forces that bind the adsorbent to the adsorbate [179]. Due to these characteristics, electrospun nanofiber membranes have been utilized to filter out heavy metal ions [180,181] and dye molecules from textile effluent [182]. The sorption potential of electrospun TPU and PVA nanofiber membranes was assessed by Akduman et al. These nanofiber membranes’ large surface areas per unit volume make them perfectly suited for the physical adsorption-based removal of certain materials. The hydrophobic structure of TPU nanofiber membranes, however, resulted in relatively low adsorptions. However, PVA nanofiber membranes, in particular those that were BTCA cross-linked, performed well when it came to the sorption of the dye Reactive Red 141. The highest possible sorption capacity was 88.31 mg/g. The sorption capacity was reduced, nevertheless, as the heat setting temperature rose from 110 °C to 130 °C. The membranes’ volume was also extremely low following the drying process and adsorption process. Thus, these materials might offer a new method for removing colour molecules from textile effluent (Table 8) [183].

## 6. Conclusions and Future Perspectives

Freshwater is obviously necessary for all living things, and if wastewater is properly treated, it can also be used again. Azo dyes are the primary source of wastewater produced by the textile sector. This calls for the necessity for an efficient azo dye wastewater treatment. Various nanomaterials were described in this review paper for the removal of azo dye from wastewater. The nanoreductive degradation by metallic amorphous or nanocrystalline alloys favored azo bond cleavage. There were also some parameters that influenced treatment results such as the pH, temperature, and dye-nanomaterial dossage. Likewise, catalysis processes with nanomaterials based on photocatalysis (including heterojunction materials), electrocatalysis, or Fenton catalysis were an alternative. One of the remaining problems after azo dye degradation was related to the formation of aromatic compounds. In order to remove these amines or phenoles, but also as an option to eliminate directly the azo molecules, one option is the use of nanosorbents. In this review, we provided scientific literature about some nanoadsorbents, which were metal oxide-based, carbon-based (including graphene), and silica-based. Furthermore, we discussed the use of nanomembranes to separate water pollutants using solution diffusion and/or size reduction.

The majority of research has been performed at a small scale in laboratories. These methods need to be scaled up to the industry scale. In conclusion, hotspots for scientific research could include real-time monitoring, material toxicity, risk analysis, mechanism elucidation, the potential for reuse, fouling problems, continuous flow operations, a lack of commercialization, and the pursuit of new-generation versatile nanomaterials, among others.

The most thoroughly studied nanomaterials include metal oxide NPs, such as TiO_2_ and ZnO, CNTs, and nanocomposites. There was also a thorough explanation of how they were used in the treatment of wastewater and water. Nanomaterials have a lot of potential for cleaning water and wastewater, given how quickly they are being created and deployed. The difficulties caused by nanomaterials must be resolved through additional research. Only a few types of nanomaterial have seen widespread commercial use. The economic effectiveness of nanomaterials should be the main topic of future research. With the extensive and regular usage of nanomaterials in water and sewage systems, the likelihood of the toxicity of nanomaterials affecting the environment and human health is also increasing. There is evidence that certain nanomaterials can have detrimental effects on the environment and human health. However, the present recommendations for assessing the dangers of nanomaterials are insufficient and weak. Nanomaterials’ toxicity needs to be thoroughly assessed before they can be used in practical applications. Additionally, there are numerous obstacles to overcome before universal or acknowledged standards are created for calculating and evaluating nanoparticles in water and sewage systems. As each nanomaterial performs differently, it is challenging to select those that warrant additional research and development. Future assessments of nanoparticles’ efficacy in treating water and wastewater will be necessary.

## Figures and Tables

**Table 1:** List of some nanoreductive materials used in the removal of organic pollutants.

Alloy Name	Route of Synthesis	Organic Pollutants	Pollutant Concentration	Alloy Dose	pH	Time	Removal	Ref.
Zerovalent iron	Liquid phase reduction method	Methyl orange	100 mg/L	0.5 g/L	4	60 min	100%	[56]
Nanozerovalent iron	Chemically synthesized	Acid black 24	100 mg/L	0.1647 g/L	6.7	80 min	80%	[57]
Nanoscale zerovalent iron	Chemically synthesized	Methylene blue	70 mg/L	10 g/L	7	30 min	72.1%	[58]
Iron-based nanoparticles	Green synthesized	Orange II	5 mg/L	10 mg/L	3	60 min	91.12%	[59]
Nanoscale zerovalent iron	Liquid phase reduction method	Basic yellow 28	100 mg/L	2 g/L	2	15 min	99.2%	[60]
Zerovalent iron powder	Commercial	Reactive black 5	100 mg/L	0.5 g/L	3	120 min	100%	[61]
Copper nanoparticles	Hydrodynamic cavitation	Methyl orange	10 mg/L	40 mg/L	3	20 min	83%	[62]
Zerovalent copper nanoparticles	Chemical reduction	Reactive blue 4	15 mg/L	1 g/L	3	10 min	90%	[63]
Zerovalent cobalt nanoparticles	Galvanic replacement method	Methyl orange	100 mg/L	0.5 g/L	2.5	4 min	99%	[52]
AlFeMnTiCr	Ball milled	Direct blue 6	200 mg/L	1 g/L	7	11 min	100%	[13]
AlCrFeMnMg	Ball milled	Direct blue 6	200 mg/L	0.5 g/L	7	6 min	100%	[39]
Mn–Al	Ball milled	Reactive black 5	40 mg/L	2.5 g/L	3	20 min	100%	[49]
Mn–Al	Ball milled	Orange II	40 mg/L	2.5 g/L	3	30 min	100%	[53]
Mn–Al	Ball milled	Brilliant green	150 mg/L	2.5 g/L	3	75 min	100%	[54]
MnAlFe	Ball milled	Reactive black 5	40 mg/L	2.5 g/L	3	5 min	100%	[55]
Ca–Al	Ball milled	Reactive black 5	40 mg	1 g/L	6	1 min	100%	[23]

**Table 2:** Azo dye degradation with amorphous alloys.

Alloy Name	Route of Synthesis	Organic Pollutants	Pollutant Concentration	Alloy Dose	pH	Time	Removal	Ref.
Fe–Si–B amorphous ribbon	Melt spinning	Rhodamine B	20 mg/L	0.5 g/L	3	10 min	100%	[67]
Fe-Si-B-Nb amourphousribbon	Melt spinning	Direct blue 15	100 mg/L	0.03 g/L	-	60 min	100%	[68]
Fe–Si–B–Cu–Nb amourphousribbon	Melt spinning	Brilliant red 3B-A	20 ppm	2 g/L	2	10 min	90%	[69]
Fe–B–Si–Y	Melt spinning	Methyl orange	20 mg/L	4 g/L	2	10 min	92%	[70]
Mg–Cu–Y	Melt spinning + mechanical milling	Direct blue 6	0.02 g/L	1.2 g/L	-	8 min	100%	[45]
Mg–Zn–Ca	Mechanical milling	Congo red	200 ppm	4 g/L	6.7	120 min	100%	[71]
Co–Si–B	Melt spinning + mechanical milling	Acid orange II	0.2 g/L	6 g/L	3	2 min	100%	[66]

**Table 8:** Azo dye degradation with nanomembranes.

Nanomembranes	Organic Pollutants	References
Chitosan	Methyl viologen, methylene blue, methyl orange, orange G, rose bengal, brilliant blue and methyl red	[184]
Nanofiltration surfactant (NFS)	Methyl violet, methyl blue and acid orange 74	[185]
Polyetherimide (PEI)-based nanofiltration (NF)	Reactive red	[186]
Hydracore 10 and hydracore 50	Cibacron yellow S-3R	[187]
Polyamide NF	Anthraquinone dyes	[188]
ZrO_2_	Dimethyl formamide	[189]

## Data Availability

Data can be requested from the authors.
